# Effectiveness of hamstring stretching using a pressure biofeedback unit for 4 weeks: A randomized controlled trial

**DOI:** 10.1142/S1013702520500092

**Published:** 2020-03-05

**Authors:** Jin-Oh Ahn, Jong-Hyuck Weon, Eun-Kyung Koh, Do-Young Jung

**Affiliations:** 1 Department of KEMA Therapy, Graduate School of Humanities Industry, Joongbu University, Geumsan, South Korea; 2 Department of Physical Therapy, College of Health & Welfare, Joongbu University, Geumsan, South Korea; 3 Department of Physical Therapy, Masan University, Changwon, South Korea; 4 Department of Physical Therapy, College of College of Health & Welfare, Joongbu University, Geumsan, South Korea; ptsports@joongbu.ac.kr

**Keywords:** Stretching, hamstrings, pressure biofeedback unit, active knee extension test

## Abstract

**Background:**

Stretching and length test of hamstring muscles have been performed commonly to manage lower back pain (LBP) in sports rehabilitation. Previous literatures addressed that stretching techniques and length test of hamstring muscles should be performed with the pelvic maintained in an anterior tilt position. However, there is no study to determine the effectiveness of pressure biofeedback unit (PBU) to maintain in anterior pelvic tilting (APT) on length test and stretching of hamstring muscles.

**Objective:**

To determine the effectiveness of hamstring muscles stretching using a PBU.

**Methods:**

Forty participants with shortness of hamstrings randomized into two groups. Participants performed the active knee extension (AKE) stretching without (control group) or with PBU (intervention group) for four weeks. AKE tests without and with PBU were administered three times before and after hamstrings stretching by each group.

**Results:**

The AKE test without PBU showed a significant main effect of time (p<0.01) but not of group (p=0.55) on the AKE angle. The AKE test with PBU showed a significant increase in the AKE angle in the post-intervention compared to the pre-intervention assessments in both groups (p<0.01). The difference of AKE angle between the pre- and post-intervention results was significantly greater in the intervention group than in the control group (p<0.01).

**Conclusion:**

We recommend the use of a PBU to maintain the pelvic anterior tilting position when performing the AKE test or AKE stretching.

## Introduction

A short hamstring is a common risk factor for lower back pain (LBP).^1,2,3,4^ Because hamstrings cross the hip, attaching proximally to the ischial tuberosity, the shortness of the hamstring muscles influences the pelvis posture.^5^ Indeed, a short hamstring may limit hip flexion, leading to compensation by tilting the pelvis in a posterior direction, which causes excessive lumbar motion during dynamic activities such as forward bending, consequently induces the LBP.^6,7^ Thus, lengthening the hamstrings may allow for greater motion in the hips and change the lumbopelvic rhythm to reduce the load on the lumbar spine.

In clinical and sports-related settings, many stretching techniques, such as static stretching, ballistic stretching, and proprioceptive neuromuscular facilitation (PNF) techniques are commonly used to improve the flexibility of the hamstring muscles in patients with LBP.^8,9,10,11^ PNF stretching uses the theories of autogenic and reciprocal inhibition to “relax” the muscle before the stretch. The hamstrings anatomically originate at the ischial tuberosity and is then inserted into the proximal tibial and fibular head.^12,13^ Because of this proximal attachment, a posterior pelvic tilt (PPT) can be used during stretching to compensate for a short hamstring.^14^ Then, it is also important to stabilize the pelvis not to increase the load of lumbar spine during stretching regardless of the stretching technique applied.

Previous researches suggested that stretching techniques for the hamstring muscles should be performed not to tilt the pelvis posteriorly.^15,16^ Sullivan *et al.* reported that although there was no significant difference in hamstrings flexibility between static and PNF stretching groups, the anterior pelvic tilting (APT) group was significantly more effective in increasing the length of their hamstring muscles than the PPT group.^16^ Then, they suggested that the APT position had a more pronounced influence than the stretching technique on the increase in the flexibility of the hamstring muscles. In this experiment, verbal instructions were provided and all stretching sessions were performed under direct supervision to maintain the pelvic tilting posture of each group. To control lumbopelvic motion during lower limb movements, clinicians commonly use several feedback tools such as tactile feedback involving touch, verbal corrections, visual feedback, and a pressure biofeedback unit (PBU).^17^ PBUs have used in clinical practice for self-monitoring lumbopelvic movement during lower limb movements. However, although many studies have demonstrated the effectiveness of a PBU for specific muscle contraction or lumbopelvic stabilization exercises, no study has investigated the effectiveness of this device for maintaining the APT position during hamstrings stretching.

Also, the pelvic position should be considered when measuring the length of the hamstring muscles. Herrington assessed the effect of the two extreme pelvic positions (maximal APT and PPT using a PBU) on the active knee extension (AKE) angle during an AKE test.^6^ The results showed that the AKE angle in the PPT position was significantly greater than that in the APT position. However, no study has assessed the length test of hamstrings with APT position using the PBU after hamstrings stretching. In this study, AKE test with PBU was performed using the PBU for maintaining the anterior pelvic tilt position. Therefore, the purpose of this study was to compare the lengths of the hamstring muscles in AKE test without and with PBU between control (AKE stretching without PBU) and intervention (AKE stretching with PBU) groups in participants with short hamstrings.

## Methods

A repeated-measures, single-blinded randomized study was conducted to determine the effect of AKE stretching with PBU on the length of hamstring muscles through the AKE test. This study was reported according to the CONSORT 2010 checklist. The purpose and protocol of this study were explained to each participant, and a signed informed consent form was obtained prior to participation. This study was approved by the Institute Review Board (IRB) of 0000000 University (JIRB-2016082401-02-160908).

## Participants

**Fig. 1.**
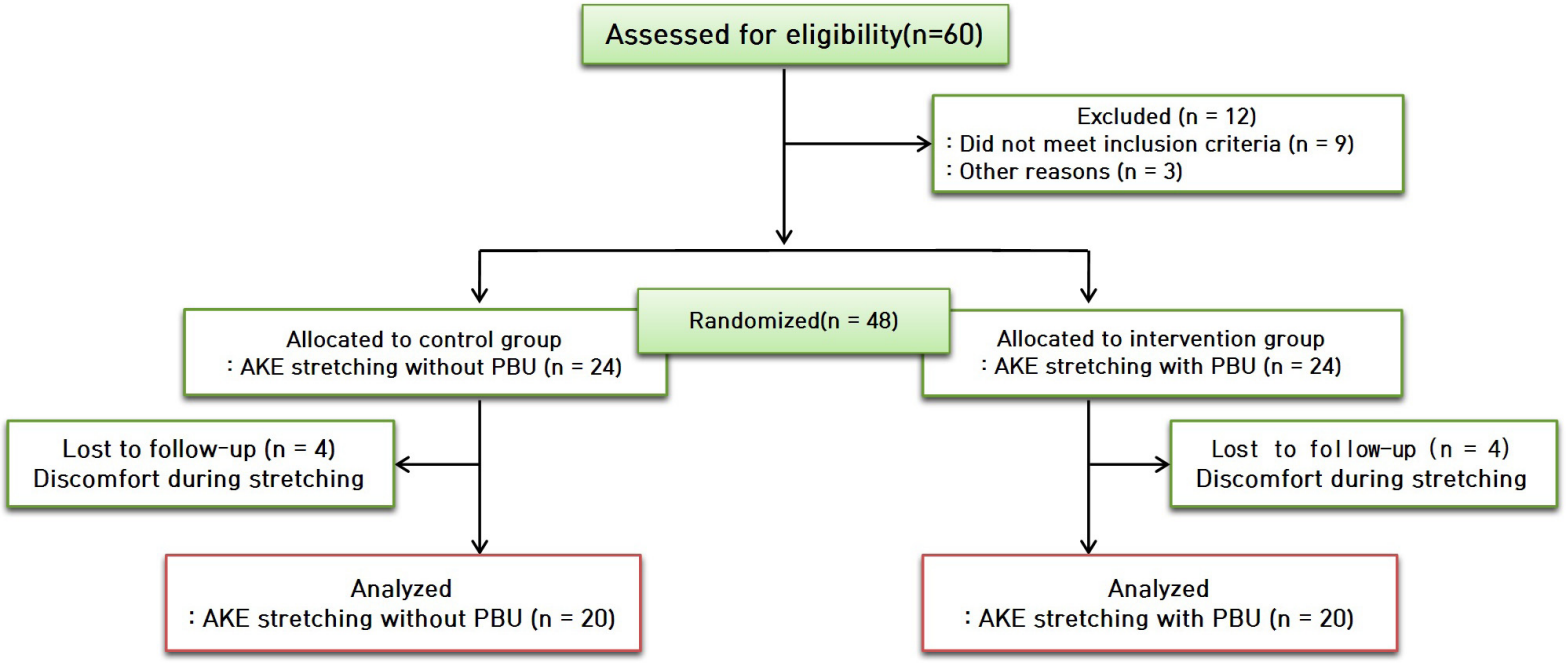
CONSORT flow diagram.

Sixty individuals volunteered to participate in this study. Forty-eight volunteers with hamstrings shorter than 70^∘^ bilaterally, as measured by the AKE test, met criteria for participation and began the study.^6^ Previous studies reported significant changes between group in length of hamstrings with sample sizes of 20 or less in each group.^18,19,20^ Based on the previous studies, a sample size of 20 in each group will yield adequate power. To allow for possible dropout, 24 participants were assigned for each group. Forty (20 males, 20 females) participants completed the study, and eight participants did not, due to pain or discomfort during stretching (Fig. 1). The following exclusion criteria were applied: (1) current or recent (last 3 months) LBP (2) history of lower limb pain or previous hamstrings injury or (3) current or recent (last 3 months) participation in a specific program designed to lengthen the hamstrings.

Each participant was randomly assigned to one of the two groups: hamstrings stretching without a PBU (control group: age 22.5±3.4 years, height 168.6±7.9 cm, weight 64.3±10.4 kg) and with a PBU (intervention group: age 26.7±3.6 years, height 168.9±7.6 cm, weight 62.4±9.6 kg). A randomized allocation list was created with a random number generator using eight blocks of six participants by a non-involved person. Allocations were sequentially numbered and placed in opaque envelopes to ensure concealed allocation. After participant enrollment was completed, the envelope was opened by the physical therapist. Participants were blinded to avoid watching the other stretching program.

## Instrumentations

The AKE test was used to determine the length of the hamstring muscles using an electrical inclinometer (Dualer IQ, J-Tech Medical, Midvale, UT, USA) as this test showed high reliability in some previous researches.^21,22,23^ A PBU (Chattanooga Group, Inc, Hixson, TN, USA) was used to maintain the anterior pelvic tilt position during AKE test or stretching, by monitoring the pressure of airbag. In this study, the AKE test was performed without and with PBU both before and after the intervention.

### Outcome measures

Both experimenters were physical therapists. An experimenter trained and supervised the participants, who had nine years of experience in orthopedic physical therapy and had used to asses lumbopelvic movement during the lower limb movement test using a PBU. Another experimenter recorded the AKE angle while performing the AKE test, who was blinded to group allocation of participants. Data collection took place at research laboratory of university and private physiotherapy clinic from January to April 2017.

**Fig. 2.**
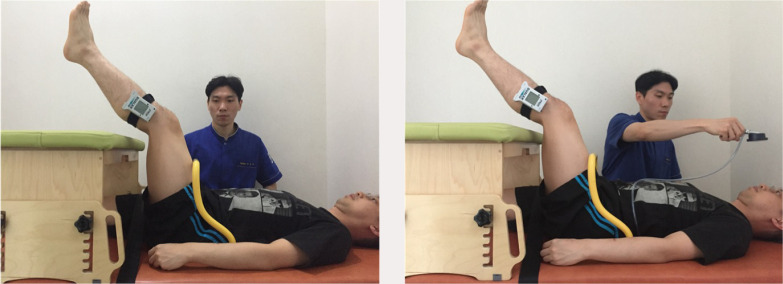
AKE test without PBU (a) and with PBU (b).

Measurements were taken with participants in a supine position on a standard treatment table with their hip and knee flexed at 90^∘^ and their anterior thigh touching the cross-bar to maintain the flexed hip. For comfort, participants placed their left leg on a wooden table to maintain the flexion in the knee at 90^∘^ before measurement. The inclinometer was attached below the fibular head by a strap. The 90^∘^ knee flexion in horizontal position was reset as 0^∘^ at the starting position (Fig. 2). Participants kept their feet relaxed without ankle dorsiflexion during the AKE test. To perform the AKE test without PBU, participants were instructed to extend their leg as much as possible, regardless of pelvic movement, while keeping their thigh against the bar; the end position was then held for 5 s. During the hold time, an experimenter recorded the AKE angle (Fig. 2(a)).

To perform the AKE test with PBU, an airbag of PBU was placed under the lumbar spine (L1–S1) centered at L3 spinous process above L4, which was palpated in middle from tops of left and right iliac crests. Each participant was asked to maximally anterior tilt the pelvis with their left hip and knee flexed at 90^∘^ and straightened right leg, “Hollow the lower back off the table as much as possible and hold it.” Then the airbag was inflated to a base pressure of 40 mmHg until it filled the available lordotic space.^17^ Keeping the hip bent and the pelvic anterior tilting (no pressure change), the participants actively extended the testing leg as far as possible while keeping their thigh against the bar; the end position was then held for 5 s. During the hold time, an experimenter recorded the AKE angle (Fig. 2(b)).

If the participant moved away from the bar, he/she was instructed to flex the knee until his/her thigh touched the bar again, and the AKE angle was determined. To eliminate the stretch effect, the participants rested for 5 min between AKE tests without and with PBU. The order of testing was carried out in block fashion with even-numbered participants starting the AKE test without PBU and odd-numbered participants the AKE test with PBU. The AKE tests without and with PBU were administered three times in each group before and after four weeks of the study period.

## Interventions

**Fig. 3.**
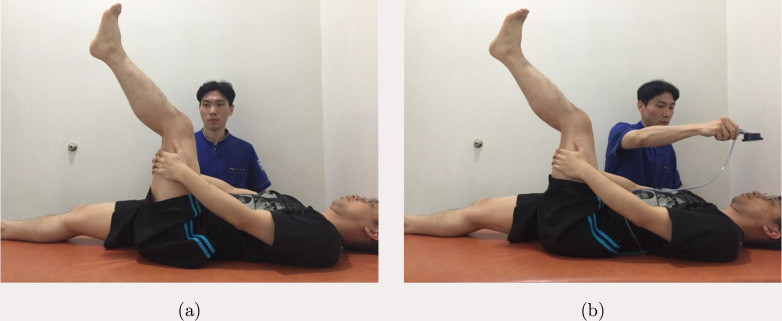
AKE stretching without PBU (a) and with PBU (b).

Participants assumed a supine position to perform the static AKE stretching to increase the flexibility of their hamstring muscles (Fig. 3). All participants actively stretched five days per week for four weeks while they were supervised by a physical therapist trained to stretch the hamstring muscles using a PBU. Each group stretched for a total of 100 s in each session and held the stretch for 10 s, for 10 repetitions, with a 10 s rest between sets. The training session lasted approximately 4 min.

Participants in the control group (AKE stretching without PBU) assumed a supine position with their hip and knee in 90–90 degrees of flexion and their foot in a relaxed position as the starting position; they then slowly extended their knee until maximum resistance was encountered. During the relaxation periods, participants were returned to the starting position. The physical therapist ensured that the stretch did not cause any pain (Fig. 3(a)).

In intervention group (AKE stretching with PBU), prior to stretching, the airbag of the PBU was placed under the lumbar spine of participants in supine position with their hip and knee in 90–90 degrees of flexion. As mentioned in outcome measure section, the airbag was then inflated to 40 mmHg in maximal anterior pelvic tilting, and participants slowly extended their knee until maximum resistance was encountered. During the stretching maneuvers, the participants were asked to maintain the pressure at 40 mmHg by viewing the pressure gage (Fig. 3(b)). On the first day of the experiment, participants in each group were familiarized with AKE stretching without or with PBU for approximately 5 min. We asked participants to complete a weekly log of the adherence to the stretching intervention.

### Statistical analysis

Independent-sample t tests were used to compare the baseline of AKE angles between the control and intervention groups. Intra-class correlation coefficients (ICCs) were used to determine the intra-rater reliability for AKE tests without and with PBU using an electronic inclinometer. The ICC${}_{3,1}$ model was computed to test the intra-rater reliability from the three repeated measurements of the AKE angle of AKE tests without and with PBU for all the participants at baseline before the intervention.

We used the averaged data from three repeated measurements of the AKE angle made by the AKE tests without and with PBU before and after the interventions. A two-way mixed-model analysis of variance (ANOVA) was used to determine the effect of time and group on the AKE angle in the AKE tests without and with PBU. When a significant time by group interaction was found, *post hoc* paired t tests were performed. Independent-sample t tests were used to compare the mean differences in the AKE angles of the control and intervention groups. To reduce the type I error rate, statistical significance for the *post hoc* paired t tests was set at α=0.01. SPSS Ver. 15.0 for Windows (SPSS, Inc., Chicago, IL, USA) was used for data analysis.

## Results

**Fig. 4.**
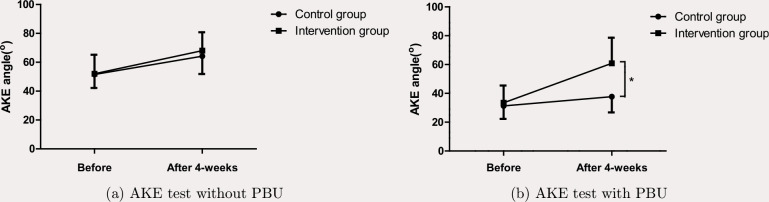
AKE angles in AKE tests without (a) and with PBU (b) before and after interventions. *Note*: *p<0.01 significance of difference in the pre- and post-intervention measurements between control and intervention groups.

The control and intervention group did not differ significantly in baseline of the AKE angle in the AKE tests without and with PBU (p>0.05). We found good intra-rater reliability for the AKE angle (AKE test without PBU: ICC${}_{3,1}=0.97$, 95% CI: 0.95–0.98; AKE test with PBU: ICC${}_{3,1}=0.96$, 95% CI: 0.93–0.98). The two-way mixed ANOVA showed a significant main effect of time in the AKE test without PBU ($F_{1,38}=196.5$, p<0.01); however, there was no significant main effect of group ($F_{1,38}=0.4$, p=0.55) on the AKE angle (Table 1) (Fig. 4).

**Table 1.** AKE angle of AKE test without PBU variations of Groups and Time.

	Intervention		Within-group difference^b^	Between-group difference	
Group	Before	After 4-weeks	(95%CI)	(95%CI)	p*
Control	$51.56\pm 9.40^{\text{a}}$	64.21±12.32	12.65 (11.55–14.94)	3.27 (0.86–7.40)	0.117
Intervention	52.12±13.22	68.04±12.74	15.92 (3.66–19.51)		

*Notes*: ^a^Mean ± SD; ^b^Within-group differences are calculated by subtracting value in post-intervention from value in post-intervention. *The p value refers to the between-group difference in angle of AKE between control and intervention groups.

In the AKE test with PBU, significant time by group interactions was found for the AKE angle ($F_{1,38}=57.7$, p<0.01). The results of the *post hoc* paired t test showed that the post-intervention AKE angle was significantly increased compared to that of the pre-intervention angle in the control (difference: 6.4^∘^, 95% CI: 3.7–9.2^∘^, p<0.01) and intervention (difference: 27.4^∘^, 95% CI: 22.3–32.5^∘^, p<0.01) groups (Table 2) (Fig. 4). An independent t test indicated that the difference between the pre- and post-intervention measurements was significantly greater in the intervention group than in the control group (between-group difference $=21.0^{\circ }$, p<0.01) (Table 2).

**Table 2.** AKE angle of AKE test with PBU of Groups and Time.

	Intervention		Within-group difference^b^	Between-group difference	
Group	Before	After 4-weeks	(95%CI)	(95%CI)	p*
Control	$31.26\pm 8.95^{\text{a}}$	37.68±10.91	6.42.(3.66–9.18)	21.00 (15.34–26.59)	0.000
Intervention	33.49±12.21	60.90±17.70	27.42(22.33–32.50)		

*Notes*: ^a^Mean ± SD; ^b^Within-group differences are calculated by subtracting value in post-intervention from value in post-intervention; *The p value refers to the between-group difference in angle of AKE between control and intervention groups.

## Discussion

In this study, we performed the AKE test to assess the length of the hamstring muscles. The intra-rater reliability for the measurement of the AKE angle was very good. Herrington reported that the mean difference in the AKE angle between the APT and PPT positions was 13.4^∘^ and noted that the position of the pelvis should be taken into consideration when measuring the length of the hamstrings because the length of this muscle influences the position of the pelvis.^6^ Thus, after four weeks of hamstrings stretching, we initially employed the AKE test using a PBU to maintain the pelvic anterior tilting. Compared to the results of a previous study,^6^ our data showed that the mean differences in the AKE angle between the AKE tests without and with PBU were 20.3^∘^ and 17.7^∘^ greater in the control group and intervention groups, respectively.^6^ In a previous study, a PBU inflated slightly was placed under the lumbar spine in the presence of a maximum PPT in the 90^∘^ hip position.^6^ Then, the knee was extended while the pressure gauge of the PBU was monitored to maintain the pressure. In contrast, the participants in our study were instructed to extend their leg as far as possible, regardless of their pelvic movement, during the AKE test without PBU. We inferred that the AKE angle in our study was greater than that in the previous study owing to greater compensatory PPT movement for a short hamstring during the AKE test without PBU.

In the AKE test with PBU, the AKE stretching using the PBU was more effective in increasing the AKE angle, as compared to the AKE stretching performed without a PBU (mean difference $=6.4^{\circ }$). However, this study also found no significant difference between the groups in the AKE angles in the AKE test without PBU (mean difference $=3.3^{\circ }$). These results indicate that the AKE test without PBU did not detect the different changes in the lengths of the hamstring muscles in the control and intervention groups after hamstrings stretching. Therefore, we recommend maintaining the pelvic anterior tilting position using a PBU when performing the AKE test or AKE stretching for length test and stretching of the hamstring muscles.

In the AKE test without PBU, the average AKE angle in the post-intervention assessment was significantly increased, by 12.7^∘^ in the control group and by 15.9^∘^ in the intervention group, compared to the comparable figures in the pre-intervention assessment. Four previous studies examining the effect of static hamstrings stretching using the AKE test without PBU reported improvements of 8.8^∘,24^ 9.1${}^{\circ },$^25^ 7.2^∘,26^ and 9.2^∘^^16^ in the AKE angle. Because both positions during the length test and the stretching maneuver were the same in our study, the increase in the AKE angle after AKE stretching would be expected to be greater in our study than in previous studies. Indeed, two previous studies confirmed the functional effect on hamstrings stretching. Yoon *et al.* reported that forward-bending using a stick was effective for preventing excessive lumbar flexion and more helpful for improving hip flexion than that without stick during forward bending.^27^ However, Li *et al.* found no change in lumbar motion during forward bending after three weeks of AKE stretching.^28^ Nonetheless, no study has compared functional movement patterns between the non-functional and functional stretching. Additional research is needed to compare the effect of AKE stretching and forward-bending stretching on forward-bending movement patterns.

Of the extant research, the study performed by Sullivan *et al.* is the one most similar to this work.^16^ They reported that the mean difference of AKE angle was significantly greater in the APT group than the PPT group after two-week hamstrings stretching. However, in our study, there was no significant difference between the groups in the AKE angle of the AKE test without PBU. This inconsistency can be explained by differences in the stretching force used. In the previous study, participants stood facing a table with one heel resting on the edge of the table at approximately 90^∘^ of hip flexion; members of both groups were then instructed to anteriorly bend their trunk while maintaining the APT or PPT position. On the other hand, participants in our study were asked to assume a supine position and extend their leg with their hip in a 90^∘^ flexed position during AKE stretching. Although participants stretched until they perceived tightness but not pain in both the previous and our research, stretching in an open versus a closed kinetic chain are associated with different stretching forces. We inferred that the greater stretching force led to the greater difference in the lengths of the hamstrings between stretching in APT and PPT positions. Further research is needed to determine the effect of stretching force (open versus closed kinetic chain) combined with pelvic position (PPT versus APT) on flexibility of hamstring muscles.

In the control group, the difference in the AKE angle in the AKE tests without and with PBU was greater after, compared with before, stretching (before stretching: 20.0^∘^ versus after stretching: 26.5^∘^). However, in the intervention group, the difference in the AKE angles between the AKE tests without and with PBU was smaller after than before stretching (before stretching: 18.6^∘^ versus after stretching: 7.1^∘^). These results reflect uncontrolled lumbopelvic movement as a result of the greater lumbar flexion and PPT movement during the conventional as opposed to during the AKE test with PBU in the control group. However, in the intervention group, the difference in the AKE angles between AKE tests without and with PBU was smaller, compared with before stretching (before stretching: 18.6^∘^ versus after stretching: 7.1^∘^). This result reflects uncontrolled lumbopelvic movement as a result of the greater lumbar flexion and PPT movements during AKE test without PBU, as opposed to during AKE test with PBU in the control group. One possible explanation for this result is that an alternative movement strategy was employed during the AKE test without PBU, which was administered to the intervention group after the AKE stretching using the PBU. Park *et al.* reported that a group who engaged in active prone knee flexion with an abdominal drawing maneuver using a PBU showed a greater reduction in their compensatory pelvic motion and LBP symptoms than did a group who stretched their rectus femoris while standing.^29^ Although we did not determine the difference in the lumbopelvic movement during the AKE tests without and with PBU, we would expect that less lumbar flexion and PPT would occur in the intervention group than control group, as a result of the altered movement pattern during the AKE test without PBU. Therefore, we postulated that AKE stretching using a PBU improved the flexibility of the hamstring muscles as well as the motor control involved in lumbopelvic stabilization.

This study has several limitations. First, although participants were blind to the experimental protocol, the investigator was aware of the identities of the control and intervention groups. The use of single blinding can lead to observation bias. Second, limitation is the lack of symptomatic individuals with LBP. Future studies should recruit patients with LBP to evaluate the effect of two different stretching approaches on the pain and function of patients. Third, we did not analyze the data using intention-to-treat. In our study, eight participants did not complete due to pain or discomfort during stretching and they were not included in data analysis. The on-protocol analysis will tend to bias results in favor of intervention effect, as those who succeed at intervention are most likely to stick with it. Finally, we did not determine the effect of a PBU on the maintenance of a pelvic position in other stretching positions. Thus, caution is needed when stretching a hamstring muscle using a PBU in either a sitting or a standing position.

## Conclusion

The difference between the pre- and post-intervention results in the AKE test with PBU was significantly greater in the group performing AKE stretching with a PBU than in the group performing AKE stretching without a PBU. We recommend the use of a PBU to maintain the pelvic anterior tilting position when performing the AKE test or AKE stretching for length test and stretching of the hamstring muscles.

## Conflict of Interest

The authors declare that there is no conflict of interest.

## Funding/Support

The authors declare that there is no funding for this study.

## Author Contributions

Jin-Oh Ahn and Do-young Jung were responsible for research conception and design of the study. Data collection and preparation for the first draft of the paper were carried by Jin-Oh Ahn and Do-young Jung. Analysis/interpretation of data, and critical revision of the paper for important intellectual content were contributed by Jong-hyuck Weon and Eun-kyung Koh. All authors read and approved the final version of the paper.
